# Comparison of Blood Loss between Open‐Box Prosthesis and Closed‐Box Prosthesis after Primary Total Knee Arthroplasty

**DOI:** 10.1111/os.12952

**Published:** 2021-03-05

**Authors:** Qi Ma, Chang‐jiao Sun, Sha Wu, Xu Cai

**Affiliations:** ^1^ Department of Orthopaedics, Beijing Tsinghua Changgung Hospital, School of Clinical Medicine Tsinghua University Beijing China

**Keywords:** Blood loss, Closed‐box prosthesis, Open‐box prosthesis, Total knee arthroplasty, Transfusion

## Abstract

**Objective:**

To compare the blood loss after procedures of primary unilateral or one‐stage bilateral total knee arthroplasty (TKA) caused by open‐box prosthesis and closed‐box prosthesis.

**Methods:**

This was a retrospective study. Patients undergoing procedures of primary TKA between January 2017 and July 2020 in our institution were assessed for eligibility for this study. Those who were diagnosed with knee osteoarthritis and underwent primary unilateral or one‐stage bilateral TKA by using PFC Sigma PS150 (closed‐box prosthesis) or Vanguard (open‐box prosthesis) knee systems and had complete data of laboratory indexes on postoperative day (POD) 1, POD 3, and POD 5 were the interested population. At last 243 patients were enrolled, among which 88 patients were classified into the unilateral closed‐box group, 66 patients into the unilateral open‐box group, 47 patients into the one‐stage bilateral closed‐box group, and 42 patients into the one‐stage bilateral open‐box group. The perioperative management and operative techniques were almost the same for each patient, except the selection of prosthesis, which was decided according to surgeon's preference. The baseline information, postoperative laboratory indexes tested on POD 1, POD 3, and POD 5 including hemoglobin, hematocrit, platelet, thrombin time (TT), prothrombin time (PT), activated partial thromboplastin time (APTT), and international normalized ratio (INR), the primary outcome measurements including the maximum decreased value of hemoglobin and the volume of total blood loss, and the secondary outcome measurements including the transfusion rate and the average transfused red blood cell (RBC) units were well compared between the open‐box group and the closed‐box group.

**Results:**

The baseline was comparable between groups, except higher preoperative levels of hemoglobin (134.43 g/L *vs* 126.51 g/L, *P* = 0.003) and hematocrit (39.92% *vs* 37.37%, *P* = 0.000) observed in the one‐stage bilateral open‐box group. The differences of postoperative coagulation function monitored by TT, PT, APTT, and INR were clinically irrelevant between groups. For patients receiving unilateral TKA, significantly higher value of decreased hemoglobin (26.06 g/L *vs* 21.05 g/L, *P* = 0.025) and significantly larger amount of total blood loss (920.34 mL *vs* 723.19 mL, *P* = 0.013) were observed in the open‐box group. For patients receiving one‐stage bilateral TKA, the open‐box prosthesis was observed to cause more hemoglobin drop (37.81 g/L *vs* 32.02 g/L, *P* = 0.071) and total blood loss (1327.26 mL *vs* 1177.42 mL, *P* = 0.247) compared to the closed‐box prosthesis, though the differences were not significant. The transfusion rate and the average transfused RBC units were not significantly different between the open‐box group and the closed‐box group no matte whether the patients were from the unilateral TKA group or from one‐stage bilateral TKA group.

**Conclusion:**

The use of open‐box prosthesis caused more hemoglobin drop and total blood loss than closed‐box prosthesis after primary unilateral or one‐stage bilateral TKA, resulting in comparable transfusion rate and average transfused RBC units between groups.

## Graphical abstract

Different characteristics between the closed‐box knee prosthesis and the open‐box knee prosthesis. The closed‐box prosthesis completely covers the whole area of cancellous bone at the distal femur, while the open‐box prosthesis leaves an uncovered area of cancellous bone at the intercondylar fossa, which may be a source of bleeding and results in more total blood loss postoperatively.

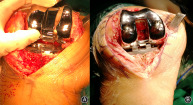

## Introduction

Total knee arthroplasty (TKA) is the most important procedure for end‐stage knee arthritis and occupies a central position in the treatment for a long period of time. For patients who previously took conservative and joint‐preserving surgical treatment but failed to achieve effective therapeutic outcomes, TKA could help them improve knee function and increase quality of life. However, postoperative blood loss is one of the most common complications after TKA and potentially leads to blood transfusion. Previous research reported the transfusion rate could be as high as 50%^1^. With the significant medical advancement over the past couple of decades, nowadays the blood transfusion rate is decreased to a range of 2% to 19%^2^.

Massive postoperative blood loss and subsequent blood transfusion jeopardize patient health *via* cardiovascular incidents, allergic reactions, hemolysis, infection, immunosuppression, or transfusion‐related liver damage^3^. Large efforts have been made to minimize the amount of postoperative bleeding and several risk factors related with higher transfusion rate are identified, including preoperative anemia, advanced age, female gender, smaller body habitus, reduced platelet function, and presence of cardiovascular comorbidity^4^, ^5^, ^6^, ^7^. More careful health care is needed for these patients in perioperative management.

Different types of knee prosthesis were also proven to be associated with postoperative blood loss after TKA. It was indicated that posterior stabilized prosthesis could cause more blood loss compared to cruciate retaining prosthesis due to the box preparation that exposed more cancellous femoral bone, though no significant difference with regard to transfusion rate was observed between them^8^. The main reason for postoperative blood loss was explained by intraoperative trauma to bone and soft tissues, and the venous sinus of the trimmed bone was referred as the main source of bleeding.

**Fig. 1:**
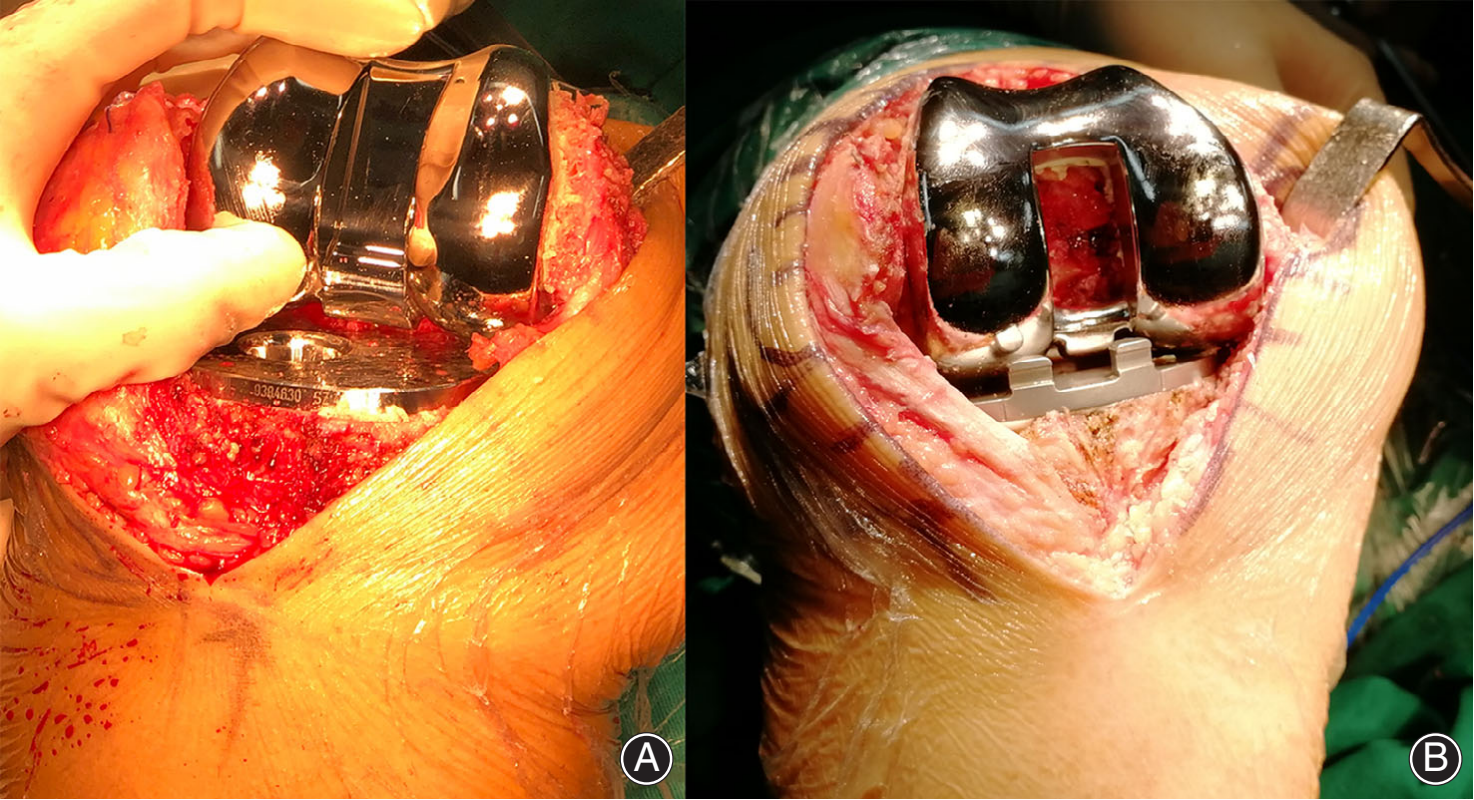
(A) The design of closed‐box prosthesis, characterized by complete covering of the whole area of cancellous bone at the distal femur. (B) The design of open‐box prosthesis, characterized by the uncovered area of cancellous bone at the intercondylar fossa, which may be a source of bleeding.

In another study, closed‐box prosthesis was compared to open‐box prosthesis in patients undergoing primary unilateral TKA and lower calculated blood loss was observed in the closed‐box group^9^. The closed‐box prosthesis is characterized by complete covering of the whole area of cancellous bone at the distal femur, while the open‐box prosthesis leaves an uncovered area of cancellous bone at the intercondylar fossa, which may be a source of significant blood loss (Fig. 1).

In this study, we retrospectively compared the difference of total blood loss (TBL) between the closed‐box prosthesis and the open‐box prosthesis after primary unilateral or one‐stage bilateral TKA, and aimed to: (i) verify the previous finding that the closed‐box prosthesis could reduce blood loss compared to the open‐box prosthesis after procedures of unilateral TKA; (ii) investigate that whether this advantage of reducing blood loss was also applicative for patients receiving primary one‐stage bilateral TKA; (iii) provide a reference for the selection of knee prosthesis.

Numerous trials were performed to find out risk factors contributing to bleeding after procedure of TKA, and corresponding interventions were carried out to prevent further blood loss and avoid postoperative transfusion. For example, the erythropoietin taken before surgery could effectively stimulate erythropoiesis and improve preoperative and postoperative hemoglobin levels and decrease transfusion rate when combined with iron therapy, helping patients get over anemia^10^. According to our protocol, if the open‐box prosthesis is proved to cause more blood loss than the closed‐box prosthesis in patients undergoing unilateral or one‐stage bilateral TKA, the uncovered areas in the intercondylar fossa in the open‐box one should be regarded as a risk factor related with further postoperative bleeding, which benefits patients by informing surgeon's selection for knee prosthesis, such as not implanting open‐box prosthesis in patient with some other risk factors of bleeding or transfusion. The difference of blood loss between open‐box prosthesis and closed‐box prosthesis did not get enough attention and relative research was limited. Except for the previously mentioned trial concerning patients with primary unilateral TKA, we could not find any other literature discussing this difference, which made it very necessary to conduct research in this area and to provide a reference and direct clinical strategy. The primary outcome measurements were the maximum postoperative decreased value of hemoglobin and the amount of TBL. The secondary outcome measurements were the transfusion rate and the average transfused red blood cell (RBC) units.

We hypothesized that the open‐box prosthesis would cause a greater drop in hemoglobin and TBL and higher transfusion rate after both primary unilateral and primary one‐stage bilateral TKA when compared to the closed‐box prosthesis.

## Materials and Methods

### Enrollment and Classification of Patients

The inclusion criteria were: (i) the patients who were diagnosed with knee osteoarthritis and underwent primary procedures of unilateral or on‐stage bilateral TKA between January 2017 and July 2020 in our hospital, and were discharged from hospital at least after postoperative day (POD) 5; (ii) posterior‐stabilized knee prosthesis with closed‐box component (PFC Sigma PS150, Depuy synthes, Warsaw, IN, USA) was implanted during operation; (iii) posterior‐stabilized knee prosthesis with open‐box component (Vanguard, Zimmer Biomet, Warsaw, IN, USA) was implanted during operation; (iv) the maximum decreased hemoglobin, TBL, transfusion rate, and average transfused RBC units could be calculated based on clinical data; (v) a retrospective study.

The exclusion criteria were: (i) diagnosed with post‐traumatic arthritis or inflammatory arthritis; (ii) underwent revision procedures of TKA; and (iii) blood routine examination and coagulation function tests were absent before operation or on POD 1, POD 3, or POD 5.

There were a total of 374 patients screened for eligibility for this study, among which nine patients were excluded because of inflammatory arthritis, 22 patients were excluded because of revised procedures of TKA, 63 patients were excluded because laboratory indexes were absent on POD 1, POD 3, or POD 5, and 37 patients were excluded because PFC Sigma PS150 or Vanguard knee system was not used during operation. Finally, 243 patients were eligible and enrolled into this study. The selection for open‐box or closed‐box prosthesis was mainly decided by surgeon's preference and was almost a random process. According to prosthesis selection and surgical features, 88 patients were classified into the unilateral closed‐box group, 66 patients into the unilateral open‐box group, 47 patients into the one‐stage bilateral closed‐box group, and 42 patients into the one‐stage bilateral open‐box group.

### Calculation of Sample Size

A sample size analysis was performed to calculate the minimum number of patients necessary for this study. Based on our pilot study and previous literature^10^, the standard deviation of hemoglobin was assumed to be 15.0 g/L and a difference of >10.0 g/L in hemoglobin concentration was considered the minimal clinical difference. With the test of significant level as 0.05 and the power of test as 80%, the minimal sample size was calculated to be 35 per group (2‐tailed), which indicated that the number of patients enrolled in each group was sufficient for this study.

### Perioperative Management and Clinical Lab Indexes

Blood samples were collected on the day of hospital admission as a baseline. The oral anticoagulants received by patients with cardiovascular or cerebrovascular diseases were changed into low‐molecular‐weight heparin (LMWH) on the day of admission. After operation, all patients received thromboprophylaxis of LMWH according to their weights until discharge from hospital. In situations where thrombosis happened, the dose was doubled. Blood samples were examined postoperatively on POD 1, POD 3, and POD 5. Clinical lab indexes such as the levels of hemoglobin, hematocrit, platelet, and coagulation markers including thrombin time (TT), prothrombin time (PT), activated partial thromboplastin time (APTT) and international normalized ratio (INR) were recorded. Patients received treatment of tranexamic acid (TXA) at a dose of 1 g per day from POD 1 to POD 3. Indication of blood transfusion was hemoglobin value <80 g/L or patients had symptoms of anemia.

### Operative Details

**Fig. 2:**
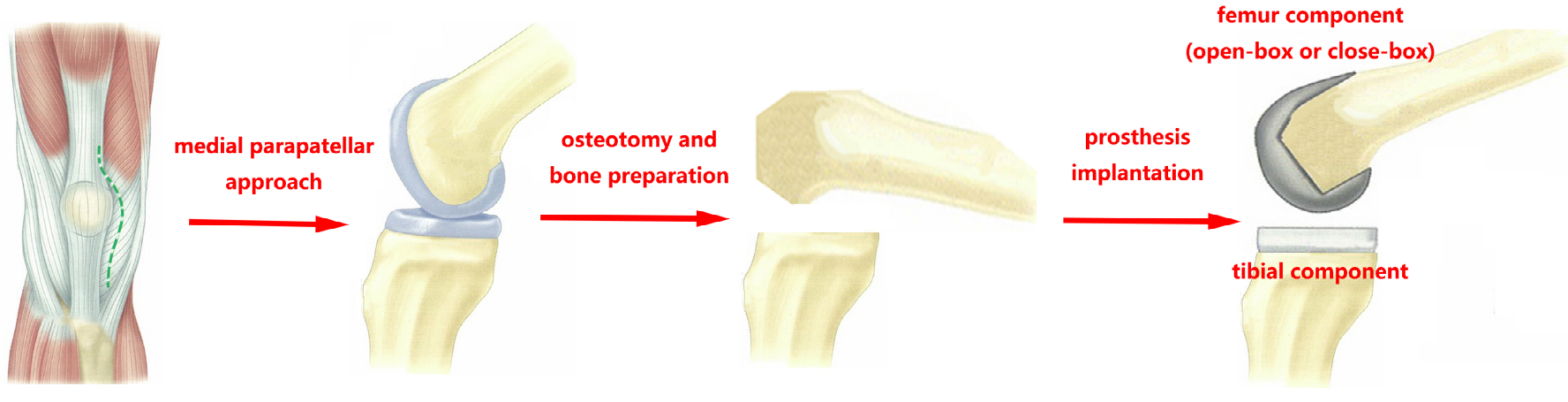
The procedures to implant prosthesis. Medial parapatellar approach was used to expose the knee joint. Measured resection technique was performed to prepare the distal femur and the proximal tibia. The open‐box or closed‐box femoral component and tibial component were subsequently implanted with cement.

Patients underwent procedures of TKA under general or spinal anesthesia according to anesthetists' preference. All surgeries were performed by one senior orthopaedic surgeon through a standard medial parapatellar approach and measured resection technique (Fig. 2). The distal femoral cut was prepared with a conventional instrument *via* an intramedullary reference while the proximal tibial cut was prepared *via* an extramedullary reference. Each prosthesis was implanted with cement. After the implantation of femoral component in the open‐box group, bone wax was used to block the uncovered areas of cancellous bone at the intercondylar fossa with the purpose of stopping further bleeding. A mixture of 80 mg of methylprednisolone, 100 mg of ropivacaine, 2 g of TXA, and 0.25 mg epinephrine was diluted with normal saline to a total volume of 50 mL, and was then injected into the articular cavity after its closure. Neither drainage tubes nor foot pumps were used postoperatively. A tourniquet controlled at 300 mm Hg pressure was used at the beginning of surgery to make a bloodless visual field. The tourniquet would be deflated if it was inflated more than 1 h and would be inflated again after a 10‐min rest and would be completely deflated after wound closure. One gram of TXA was provided intravenously 10 min prior to the initial incision.

### Outcome Measurements

#### Maximum Decreased Hemoglobin

The maximum decreased hemoglobin was the difference between the minimum postoperative hemoglobin value among those tested on POD 1, POD 3, POD 5, and the hemoglobin value tested on the day of admission. The maximum decreased hemoglobin was a parameter to show the most remarkable change in hemoglobin level before and after surgery.

#### Total Blood Loss

Patient's blood volume (PBV) was calculated as followed: PBV = K1 × height^3^ (m) + K2 × weight (kg) + K3; K1 = 0.3669, K2 = 0.03219, K3 = 0.6041 for men; and K1 = 0.3561, K2 = 0.03308, K3 = 0.1833 for women. TBL was calculated by the Gross formula: TBL = PBV × (hematocrit_pre_–hematocrit_post_)/hematocrit_ave_; hematocrit_pre_ = the hematocrit value tested on the day of admission; hematocrit_post_ = the minimum postoperative hematocrit value among those tested on POD 1, POD 3, and POD 5; hematocrit_ave_ = (hematocrit_pre_ + hematocrit_post_)/2^11^. The TBL was a parameter to directly measure the amount of bleeding caused by surgery.

#### Transfusion Rate

Transfusion rate was the proportion of patients who were transfused postoperatively because of postoperative hemoglobin level <80 g/L or complaints of symptoms of anemia. The transfusion rate was an important and conventional assessment of the severity of bleeding after surgery. Higher transfusion rates were associated with more blood loss caused by surgery.

#### Average Transfused Red Blood Cell Units

The total transfused RBC units divided by the number of all patients equaled the average transfused RBC units, which was another important assessment for bleeding after surgery.

### Statistical Analysis

Continuous variable was presented as mean and standard deviation and discrete variable as frequency and percentage. Quantitative variables including the age, height, weight, body mass index (BMI), operative duration, values of hemoglobin, hematocrit, platelet, TT, PT, APTT, INR, and the maximum decreased hemoglobin and TBL were compared by Student's *t* test. Qualitative variables including ratio of affected side, gender, type of anesthesia, and incidences of comorbidities, transfusion rate, and average transfused RBC units were compared by Pearson's chi‐square test or Fisher's exact test. All statistical analyses were performed with SPSS version 19.0 (SPSS Inc., Chicago, IL, USA). The probability of a type I error (the α) was 0.05, and a *P* value of <0.05 was considered statistically significant.

## Results

### Comparison of the Baseline

The baseline of the patients enrolled in this study was shown in Table 1.

**TABLE 1:** Baseline information

Variables	Unilateral TKA		Statistic value	*P* value	One‐stage bilateral TKA		Statistic value	*P* value
	Closed‐box group (*n* = 88)	Open‐box group (*n* = 66)			Closed‐box group (*n* = 47)	Open‐box group (*n* = 42)		
Age(yr)	66.92 ± 7.24	69.30 ± 10.26	*t* = −1.679	*0.095*	66.60 ± 7.62	66.36 ± 7.93	*t* = 0.143	*0.887*
Height (cm)	159.08 ± 6.65	159.90 ± 7.66	*t* = −0.691	*0.491*	157.79 ± 7.06	160.07 ± 6.02	*t* = −1.603	*0.113*
Weight (kg)	69.67 ± 10.65	68.39 ± 9.93	*t* = 0.831	*0.407*	69.96 ± 15.39	69.78 ± 10.34	*t* = 0.061	*0.952*
BMI (kg/m^2^)	27.56 ± 4.11	26.71 ± 3.08	*t* = 1.379	*0.170*	27.95 ± 4.76	27.24 ± 3.90	*t* = 0.742	*0.460*
Side			χ^2^ = 0.264	*0.608*			N/A	*N/A*
Left[*n* (%)]	39 (44.3)	32 (48.5)			N/A	N/A		
Right[*n* (%)]	49 (55.7)	34 (51.5)			N/A	N/A		
Gender			χ^2^ = 2.416	*0.120*			χ^2^ = 2.746	*0.098*
Male [*n* (%)]	16 (18.2)	19 (28.8)			5 (10.6)	10 (23.8)		
Female[*n* (%)]	72 (81.8)	47 (71.2)			42 (89.4)	32 (76.2)		
Comorbidity								
Hypertension [*n* (%)]	54 (61.36)	32 (48.48)	χ^2^ = 0.729	*0.393*	25 (53.19)	25 (59.52)	χ^2^ = 0.101	*0.750*
Coronary Heart disease, [*n* (%)]	11 (12.5)	3 (4.55)	χ^2^ = 2.435	*0.119*	2 (4.26)	5 (11.90)	χ^2^ = 1.526	*0.217*
Diabetes, *n* (%)	21 (23.86)	16 (24.24)	χ^2^ = 0.002	*0.966*	9 (19.15)	5 (11.90)	χ^2^ = 0.642	*0.423*
Preoperative lab index								
Hemoglobin, (g/L)	130.66 ± 12.56	130.14 ± 13.48	*t* = 0.246	*0.806*	126.51 ± 11.33	134.43 ± 12.78	*t* = −3.063	*0.003*
Hematocrit (%)	38.36 ± 3.59	38.40 ± 3.83	*t* = −0.073	*0.942*	37.37 ± 3.02	39.92 ± 3.49	*t* = −3.636	*0.000*
Platelet (×10^9^/L)	230.17 ± 71.86	217.88 ± 56.16	*t* = 1.143	*0.255*	234.96 ± 55.27	239.62 ± 52.26	*t* = −0.403	*0.688*
TT (sec)	17.97 ± 0.62	17.95 ± 0.85	*t* = 0.134	*0.894*	17.92 ± 0.78	18.14 ± 0.64	*t* = −1.471	*0.145*
PT (sec)	11.35 ± 0.84	11.67 ± 1.20	*t* = −1.967	*0.051*	11.28 ± 0.67	11.45 ± 0.62	*t* = −1.193	*0.236*
APTT (sec)	26.20 ± 4.19	26.74 ± 3.87	*t* = −0.804	*0.422*	26.33 ± 3.63	26.70 ± 3.50	*t* = −0.477	*0.635*
INR	0.98 ± 0.09	1.00 ± 0.12	*t* = −1.390	*0.166*	0.96 ± 0.06	0.98 ± 0.06	*t* = −0.966	*0.337*

BMI, body mass index.

The age, height, weight, BMI and preoperative levels of hemoglobin, hematocrit, platelet, TT, PT, APTT and INR were compared by using independent samples *t* test. The proportion of affected side and gender, and the incidences of comorbidities were compared with chi‐square test.

In the unilateral groups, the mean age in the closed‐box and the open‐box groups were 66.92 ± 7.24 and 69.30 ± 10.26 years, the mean height were 159.08 ± 6.65 and 159.90 ± 7.66 cm, the mean weight were 69.67 ± 10.65 and 68.39 ± 9.93 kg, and the mean BMI were 27.56 ± 4.11 and 26.71 ± 3.08 kg/m^2^ respectively, which were not significantly different between two groups (all *P* > 0.05). In the one‐stage bilateral groups, the mean age, height, weight, and BMI in the closed‐box and the open‐box groups were 66.60 ± 7.62 and 66.36 ± 7.93 years, 157.79 ± 7.06 and 160.07 ± 6.02 cm, 69.96 ± 15.39 and 69.78 ± 10.34 kg, and 27.95 ± 4.76 and 27.24 ± 3.90 kg/m^2^ respectively, and were not significantly different either (all *P* > 0.05).

For patients with unilateral TKA, the left (right) lower limbs receiving procedures of TKA accounted for 44.3% (55.7%) in the closed‐box group and 48.5% (51.5%) in the open‐box group, and no significant difference was found for this ratio (*P* = 0.608). The proportions of male (female) patients were 18.2% (81.8%) in the closed‐box group and 28.8% (71.2%) in the open‐box group, showing no significant difference between groups (*P* = 0.120). The incidences of comorbidities, including hypertension (61.36% *vs* 48.48%, *P* = 0.393), coronary heart disease (12.5% *vs* 4.55%, *P* = 0.119), and diabetes (23.86% *vs* 24.24%, *P* = 0.966), were not significantly different between groups either. The preoperative levels of hemoglobin (130.66 ± 12.56 g/L *vs* 130.14 ± 13.48 g/L), hematocrit (38.36% ± 3.59% *vs* 38.40% ± 3.83%), platelet (230.17 ± 71.86 ×10^9^/L *vs* 217.88 ± 56.16 × 10^9^/L), TT (17.97 ± 0.62 seconds *vs* 17.95 ± 0.85 seconds), PT (11.35 ± 0.84 seconds *vs* 11.67 ± 1.20 seconds), APTT (26.20 ± 4.19 seconds *vs* 26.74 ± 3.87 seconds), and INR (0.98 ± 0.09 *vs* 1.00 ± 0.12) were also found to be comparable between groups (all *P* > 0.05).

For patients with one‐stage bilateral TKA, the male (female) patients accounted for 10.6% (89.4%) in the closed‐box group and 23.8% (76.2%) in the open‐box group, which was not significantly different between groups (*P* = 0.098). The incidences of hypertension (53.19% *vs* 59.52%), coronary heart disease (4.26% *vs* 11.90%), and diabetes (19.15% *vs* 11.90%) were not significantly different either, with *P* values of 0.750, 0.217, and 0.423, respectively. The preoperative laboratory indexes were found comparable except for hemoglobin (126.51 ± 11.33 *vs* 134.43 ± 12.78 g/L) and hematocrit (37.37% ± 3.02% *vs* 39.92% ± 3.49%). The former was 7.92 g/L (*P* = 0.003) and the latter was 2.55% (*P* = 0.000) higher in the open‐box group compared to those in the closed‐box group, respectively.

### Comparison of Operative Details

Operative details were presented in Table 2.

**TABLE 2:** Operative information

Variables	Unilateral TKA		Statistic value	*P* value	One‐stage bilateral TKA		Statistic value	*P* value
	Closed‐box group (*n* = 88)	Open‐box group (*n* = 66)			Closed‐box group (*n* = 47)	Open‐box group (*n* = 42)		
Type of anesthesia			χ^2^ = 0.020	*0.887*			χ^2^ = 0.079	*0.779*
General anesthesia, *n* (%)	37 (42.0)	27 (40.9)			17 (36.2)	14 (33.3)		
Spinal anesthesia, *n* (%)	51 (58.0)	39 (59.1)			30 (63.8)	28 (66.7)		
Operative duration, min	111.40 ± 24.59	109.73 ± 19.35	*t* = 0.453	*0.651*	208.60 ± 31.79	208.14 ± 33.81	*t* = 0.064	*0.949*

The type of anesthesia was compared by using chi‐square test. The operative duration was compared with independent samples *t* test.

In unilateral TKA group, the percentages of patients who received general (spinal) anesthesia were 42.0% (58.0%) in the closed‐box group and 40.9% (59.1%) in the open‐box group, showing no significant difference between them (*P* = 0.887). Additionally, no significant difference was found with regard to operative duration between groups (111.40 ± 24.59 min *vs* 109.73 ± 19.35 min, *P* = 0.651).

In one‐stage bilateral TKA group, the percentages of general (spinal) anesthesia were 36.2% (63.8%) in the closed‐box group and 33.3% (66.7%) in the open‐box group, presenting no significant difference between groups (*P* = 0.779). Besides, we could not find any significant difference in operative duration between the closed‐box group and the open‐box group (208.60 ± 31.79 min *vs* 208.14 ± 33.81 min, *P* = 0.949).

### Comparison of Postoperative Lab Indexes

The comparison of postoperative clinical lab indexes was described in Table 3.

**TABLE 3:** Comparison of postoperative clinical lab indexes

Variables	Unilateral TKA		Statistic value	*P* value	One‐stage bilateral TKA		Statistic value	*P* value
	Closed‐box group (*n* = 88)	Open‐box group (*n* = 66)			Closed‐box group (*n* = 47)	Open‐box group (*n* = 42)		
Hemoglobin, g/L								
POD 1	124.21 ± 12.39	121.80 ± 12.69	*t* = 1.169	*0.244*	114.46 ± 12.10	121.31 ± 13.10	*t* = −2.522	*0.013*
POD 3	111.05 ± 12.06	104.71 ± 12.52	*t* = 2.758	*0.008*	95.91 ± 17.13	98.54 ± 14.93	*t* = −0.675	*0.502*
POD 5	107.88 ± 13.81	101.50 ± 14.13	*t* = 2.132	*0.036*	92.36 ± 14.14	94.56 ± 12.34	*t* = −0.590	*0.558*
Hematocrit, %								
POD 1	36.06 ± 3.57	35.33 ± 3.49	*t* = 1.255	*0.211*	33.51 ± 3.12	35.77 ± 3.49	*t* = −3.177	*0.002*
POD 3	32.56 ± 3.61	30.62 ± 3.41	*t* = 2.897	*0.005*	28.31 ± 4.55	29.42 ± 3.98	*t* = −1.068	*0.289*
POD 5	31.41 ± 4.10	29.75 ± 3.98	*t* = 1.936	*0.056*	27.19 ± 4.07	27.88 ± 3.25	*t* = −0.671	*0.505*
Platelet, ×10^9^/L								
POD 1	221.32 ± 70.77	216.58 ± 53.98	*t* = 0.451	*0.653*	209.74 ± 51.36	225.21 ± 46.79	*t* = −1.456	*0.149*
POD 3	195.09 ± 51.35	185.12 ± 49.55	*t* = 1.033	*0.304*	187.80 ± 47.77	189.11 ± 43.00	*t* = −0.119	*0.905*
POD 5	243.34 ± 100.49	223.80 ± 59.27	*t* = 1.076	*0.285*	227.93 ± 46.79	235.20 ± 61.50	*t* = −0.478	*0.635*
TT, sec								
POD 1	17.60 ± 0.71	17.77 ± 1.03	*t* = −1.165	*0.246*	17.41 ± 0.99	17.42 ± 0.59	*t* = −0.097	*0.923*
POD 3	16.17 ± 0.68	16.27 ± 0.74	*t* = −0.704	*0.483*	16.03 ± 0.96	15.91 ± 0.53	*t* = 0.610	*0.545*
POD 5	16.47 ± 0.68	16.62 ± 1.35	*t* = −0.599	*0.551*	16.58 ± 2.02	16.27 ± 0.73	*t* = 0.643	*0.524*
PT, sec								
POD 1	11.52 ± 0.73	11.84 ± 0.68	*t* = −2.721	*0.007*	11.63 ± 0.68	11.81 ± 0.55	*t* = −1.373	*0.173*
POD 3	11.57 ± 0.55	11.87 ± 0.71	*t* = −2.336	*0.022*	11.56 ± 0.78	11.73 ± 0.51	*t* = −0.968	*0.337*
POD 5	11.70 ± 0.95	12.30 ± 2.94	*t* = −1.236	*0.221*	11.44 ± 0.59	11.42 ± 0.54	*t* = 0.137	*0.892*
APTT, sec								
POD 1	25.23 ± 4.88	26.03 ± 4.55	*t* = −1.028	*0.306*	25.94 ± 4.34	25.64 ± 3.33	*t* = 0.361	*0.719*
POD 3	27.18 ± 4.74	28.36 ± 4.71	*t* = −1.211	*0.229*	28.63 ± 5.69	27.83 ± 4.69	*t* = 0.593	*0.555*
POD 5	27.50 ± 5.50	27.85 ± 5.28	*t* = −0.263	*0.794*	27.93 ± 4.58	26.41 ± 2.90	*t* = 1.253	*0.217*
INR								
POD 1	0.99 ± 0.07	1.01 ± 0.06	*t* = −1.805	*0.073*	1.00 ± 0.06	1.01 ± 0.06	*t* = −0.836	*0.405*
POD 3	1.00 ± 0.06	1.02 ± 0.06	*t* = −1.715	*0.090*	1.00 ± 0.06	1.01 ± 0.06	*t* = −0.809	*0.422*
POD 5	1.02 ± 0.10	1.06 ± 0.29	*t* = −0.935	*0.353*	0.99 ± 0.06	0.98 ± 0.06	*t* = 0.508	*0.614*

APTT, activated partial thromboplastin time; INR, international normalized ratio; PT, prothrombin time; Sec, seconds; TT, thrombin time.

The values of hemoglobin, hematocrit, platelet, TT, PT, APTT, INR were compared with independent samples *t* test.

Within unilateral TKA groups, we found the values of hemoglobin on POD 3 (104.71 ± 12.52 g/L), POD 5 (101.50 ± 14.13 g/L), and hematocrit on POD 3 (30.62% ± 3.41%) in the open‐box group were significantly lower than those in the closed‐box group (111.05 ± 12.06 g/L, 107.88 ± 13.81 g/L and 32.56% ± 3.61%) with a mean difference of 6.34 g/L (*P* = 0.008), 6.38 g/L (*P* = 0.036) and 1.94% (*P* = 0.005), respectively. The postoperative values of PT on POD 1 (11.84 ± 0.68 s) and POD 3 (11.87 ± 0.71 s) in the open‐box group were 0.32 s (*P* = 0.007) and 0.30 s (*P* = 0.022) significantly higher than those in the closed‐box group (11.52 ± 0.73 and 11.57 ± 0.55 s, respectively). Other postoperative indexes were not significantly different (all *P* > 0.05).

Within one‐stage bilateral TKA groups, there were significantly higher values of hemoglobin on POD 1 and hematocrit on POD 1 in the open‐box group, with a mean difference of 6.85 g/L (121.31 ± 13.10 *vs* 114.46 ± 12.10 g/L, *P* = 0.013) and 2.26% (35.77% ± 3.49% *vs* 33.51% ± 3.12%, *P* = 0.002), respectively. It was not significantly different with regard to other postoperative indexes (all *P* > 0.05).

### Comparison of Primary and Secondary Outcomes

The comparison of the primary and secondary outcome measurements was presented in Table 4.

**TABLE 4:** Comparison of primary and secondary measurement outcomes

Variables	Unilateral TKA		Statistic value	*P* value	One‐stage bilateral TKA		Statistic value	*P* value
	Closed‐box group (*n* = 88)	Open‐box group (*n* = 66)			Closed‐box group (*n* = 47)	Open‐box group (*n* = 42)		
Maximum decreased hemoglobin, g/L	21.05 ± 13.26	26.06 ± 13.78	*t* = −2.269	0.025	32.02 ± 14.96	37.81 ± 14.47	*t* = −1.829	0.071
Total blood loss, mL	723.19 ± 458.44	920.34 ± 476.06	*t* = −2.523	*0.013*	1177.42 ± 615.79	1327.26 ± 562.15	*t* = −1.167	*0.247*
Transfusion rate, *n* (%)	1 (1.14)	2 (3.03)	χ^2^ = 0.679	*0.410*	8 (17.02)	5 (11.90)	χ^2^ = 0.348	*0.555*
Total transfused RBC units	2	4	N/A	*N/A*	19.5	10	N/A	*N/A*
Average transfused RBC units	0.023	0.061	χ^2^ = 1.330	*0.249*	0.415	0.238	χ^2^ = 1.751	*0.186*

RBC, red blood cell.

The maximum decreased hemoglobin and the total blood loss were compared by using independent samples *t* test. The transfusion rate and the average transfused RBC units were compared with chi‐square test.

Within unilateral TKA groups, the maximum value of decreased hemoglobin and the volume of TBL were significantly higher in the open‐box group than those in the closed‐box group (decreased hemoglobin, 26.06 g/L *vs* 21.05 g/L, *P* = 0.025; TBL, 920.34 mL *vs* 723.19 mL, *P* = 0.013), with a difference of 5.01 g/L and 197.15 mL, respectively. For the transfusion rate and the average transfused RBC units, no significant differences were found between groups (transfusion rate, 1.14% *vs* 3.03%, *P* = 0.410; average transfused RBC units, 0.023 *vs* 0.061 units, *P* = 0.249).

Within one‐stage bilateral TKA groups, however, the maximum hemoglobin drop and TBL were not significantly different, despite higher levels of these outcome measurements observed in the open‐box group (decreased hemoglobin, 37.81 g/L *vs* 32.02 g/L, *P* = 0.071; TBL, 1327.26 mL *vs* 1177.42 mL, *P* = 0.247), with a difference of 5.79 g/L and 149.84 mL, respectively. Being similar to the results in the unilateral TKA group, the differences of the transfusion rate and the average transfused RBC units between the one‐stage bilateral TKA subgroups were not significant either (transfusion rate, 17.02% *vs* 11.90%, *P* = 0.555; average transfused RBC units, 0.415 *vs* 0.238 units, *P* = 0.186).

## Discussion

### Baseline and Postoperative Lab Indexes

It has been widely accepted that a series of risk factors were associated with increased TBL after procedures of TKA^4^, ^5^, ^6^, ^7^, ^12^, ^13^, such as the elderly, female patients, smaller BMI due to decreased hematopoietic activity, reduced coagulation function, presence of cardiovascular comorbidity, longer operative duration, general anesthesia, and anemia. It was found that a preoperative hemoglobin level of <130 g/L could increase the risk of transfusion to four to 15 times higher compared with hemoglobin above that level^4^, ^5^.

In our study, the demographic characteristics and preoperative and intraoperative details of the patients with procedures of unilateral TKA, including age, height, weight, BMI, proportion of affected side, gender ratio, incidence of comorbidities (hypertension, coronary heart disease, and diabetes in this study), proportion of general or spinal anesthesia, operative duration, and preoperative lab indexes (hemoglobin, hematocrit, platelet, TT, PT, APTT, and INR), were not significantly different between the closed‐box group and the open‐box group, showing a comparable baseline within these two subgroups. In patients undergoing simultaneous bilateral TKA, the baseline information was also comparable except for the obvious higher preoperative levels of hemoglobin and hematocrit in the open‐box group.

The postoperative coagulation function, which was represented by postoperative count of platelets, and the postoperative values of TT, PT, APTT, and INR, was found not to be significantly different between the unilateral TKA subgroups, except the PT tested on POD 1 and POD 3. These differences could be explained by the preoperative insignificantly different levels of PT, whose *P* value was near 0.05 (*P* = 0.051). However, the mean differences of PT on POD 1 and POD 3 were 0.32 s and 0.30 s, respectively. We thought these subtle differences were clinically irrelevant and would not lead to obvious differences in coagulation function on POD 1 and POD 3.

### Outcomes in Patients with Unilateral TKA

For these patients with procedures of unilateral TKA, under the premise of a comparable baseline, the levels of hemoglobin on POD 3 and POD 5 and hematocrit on POD 3 were, respectively, 6.34 g/L, 6.38 g/L, and 1.94% significantly lower in the open‐box group. Furthermore, the maximum value of decreased hemoglobin and the volume of TBL were 5.01 g/L and 197.15 mL significantly higher in the open‐box group, indicating that the open‐box prosthesis indeed caused more blood loss than the closed‐box prosthesis, which was in accordance with previous findings mentioned in the introduction. Nevertheless, this disadvantage resulted in no significant difference in the transfusion rate and the average transfused RBC units between unilateral closed‐box group and open‐box group in this study.

There had been several studies concerning postoperative blood loss caused by different types of prosthesis. Mähringer‐Kunz *et al*. announced that posterior‐stabilized prosthesis caused 46 ml more calculated blood loss than cruciate‐retaining prosthesis in a retrospective study enrolling 473 patients^8^. They assumed that the uncovered areas of cancellous bone cut by different prosthesis designs were the main reasons for this difference. However, research about the comparison of blood loss caused by closed‐box and open‐box prostheses was relatively rare. To our best knowledge, only a single related study could be found. In that prospective study, the authors recruited 228 patients undergoing unilateral TKA and concluded that open‐box prosthesis had 85.6 ml more calculated blood loss compared to closed‐box prosthesis^9^. Unfortunately, the comparability of perioperative coagulation function between groups was not clearly described in that article, leaving queries about the existence of significant difference in perioperative bleeding tendency. In our study, the perioperative coagulation function between unilateral TKA subgroups was clearly described by comparing levels of platelet, TT, PT, APTT, and INR, resulting in comparable coagulation function and making the results more accurate while minimizing the bias derived from difference of coagulation function.

### Outcomes in Patients with One‐stage Bilateral TKA

For these patients with procedures of one‐stage bilateral TKA, with the premise of comparable perioperative coagulation function and demographics, the levels of hemoglobin on POD 1 and hematocrit on POD 1 were 6.85 g/L and 2.26% higher, respectively, in the open‐box group. However, these significant differences were not precise and could not reflect the truth because of the significantly higher levels of hemoglobin and hematocrit in the open‐box group before operation. As for the primary outcomes, the maximum hemoglobin drop and TBL were 5.79 g/L and 149.84 mL higher, respectively, in the open‐box group compared to those in the closed‐box group, though the differences were not statistically significant. With regard to the secondary outcomes, the differences of transfusion rate and average transfused RBC units could not be found significant between subgroups either. We believed that the open‐box prosthesis caused more blood loss than the closed‐box one, but this disadvantage seemed to be minimized in patients with one‐stage bilateral TKA. It was worth noting that in the one‐stage bilateral TKA groups, the mean preoperative value of hemoglobin in the open‐box group was significantly higher than that in the closed‐box group (134.43 ± 12.78 g/L *vs* 126.51± 11.33 g/L, *P* = 0.003).

As mentioned in previous section, the preoperative hemoglobin less than 130 g/L was a risk factor of transfusion^4^, ^5^. In our case, the mean preoperative value of hemoglobin in the closed‐box group was less than 130 g/L (126.51 ± 11.33 g/L), while in the open‐box group the value was more than 130 g/L (134.43 ± 12.78 g/L). Therefore, we thought that the patients from the closed‐box group were more likely to lose blood and be transfused postoperatively than those from the open‐box group. This influence might narrow the difference in TBL between the two groups and make this difference not statistically significant. As far as we know, there has not been any previous study focusing on the comparison of blood loss caused by open‐box and closed‐box prosthesis in patients undergoing one‐stage bilateral TKA. Our study seemed to be the first to propose a perspective on this question.

### Strategies to Stop Further Bleeding

Several methods had been taken to reduce bleeding. During operation, we used bone wax to block the uncovered areas of cancellous bone at the intercondylar fossa in patients with open‐box prosthesis. There had been previous work reporting significant effect of reducing TBL in patients undergoing unilateral TKA after applying bone wax onto uncovered bone around prosthesis intraoperatively^14^.

Besides, we also used tourniquet with pressure controlled at 300 mmHg and intraoperative and postoperative TXA (during operation, intravenous 1 g plus topical 2 g for unilateral TKA and intravenous 1 g plus topical 4 g for bilateral TKA; after operation, intravenous 3 g in total for all TKA) to stop further bleeding. The effect of reducing blood loss by tourniquet was still under debate. A recently published meta‐analysis pointed out that using tourniquet could significantly decrease intraoperative blood loss and calculated blood loss, but did not significantly decrease postoperative blood loss, total blood loss, or transfusion rate^15^. Despite the queries on the effect of hemostasis, other questions like what is the optimal pressure, what is the most appropriate time duration for application of tourniquet, and when should the tourniquet be inflated or deflated, are all controversial^16^, ^17^.

As for the application of TXA, numerous studies have proven its effect of hemostasis in patients with procedures of TKA due to the inhibiting of fibrinolysis, no matter whether it was taken orally, intravenously, or topically^18^, ^19^, ^20^. The administration of TXA in a dose of 1 g per day intravenously on the first three postoperative days has been a conventional method for reducing blood loss in our institution for a long time. It was widely accepted that fibrinolysis peaked 6 h after total joint arthroplasty and was maintained about 18 h postoperatively^21^. So it seemed that the first 1 g TXA used on the first postoperative day played a major role in reducing blood loss. Though the effect of our conventional method of administering TXA has not been researched in previous work, a recently published study supported our method indirectly^22^. In that literature, the patients in group A received intravenous TXA at 20 mg/kg 10 min before the surgery and 3 h postoperatively, and then oral 1 g TXA from POD 1 to POD 14, and the patients in group B received the same intravenous TXA, and then oral 1 g placebo from POD 1 to POD 14. The minimum postoperative values of hemoglobin and hematocrit appeared on POD 3, which were chosen to calculate the TBL. The results presented that the TBL in group A was significantly lower than that in group B (671.7 mL *vs* 915.8 mL, *P* = 0.001), proving the oral TXA on the first 3 postoperative days could reduce blood loss effectively. In our case, on the first 3 postoperative days the TXA was administered not orally, but intravenously, and we believe our method would have a similar hemostasis effect, just like what was mentioned in that article^22^.

Furthermore, a cocktail therapy consisting of normal saline, methylprednisolone, ropivacaine, TXA, and epinephrine was applied intra‐articularly after the closure of the articular cavity. TXA could reduce blood loss as mentioned above. Ropivacaine is a nerve blocker even at a low concentration. Methylprednisolone was helpful because of its anti‐inflammatory analgesic effect. Epinephrine can prolong the action time of the drug by contracting the anterior sphincter of arteriole and capillaries. The hemostatic effect of this mixture had been confirmed in previous work^23^.

In our study, the hemostatic combination of tourniquet, TXA, and cocktail was performed in all patients and an extra application of bone wax was added into the open‐box groups. According to our results, the increased TBL and more hemoglobin drop observed in the open‐box groups suggested to us that the union of all these hemostatic methods still could not effectively stop further bleeding caused by the open‐box characteristic.

### Limitations

There were some limitations in our findings, beginning with the retrospective nature of this study. Also, the sample size was not large enough so that some significant differences might not be well distinguished, such as the differences in transfusion rate and average transfused RBC units. It had been widely accepted that controlled intraoperative hypotension was a means for reducing blood loss and transfusions^24^. Unfortunately, the patients’ intraoperative blood pressure was not documented so that we could not analyze the influence produced by the difference of intraoperative blood pressure on our results. Last, our comparison was performed between only two types of prosthesis and by a single surgeon, which could be regarded as another limitation due to a lack of generality.

## Conclusions

Use of open‐box prosthesis caused more hemoglobin drop and TBL than closed‐box prosthesis after both primary unilateral and one‐stage bilateral procedures of TKA. Nevertheless, the application of either type of prosthesis resulted in comparable transfusion rate and average transfused RBC units.
